# Polyextremophilic Bacteria from High Altitude Andean Lakes: Arsenic Resistance Profiles and Biofilm Production

**DOI:** 10.1155/2019/1231975

**Published:** 2019-02-21

**Authors:** Federico Zannier, Luciano Raúl Portero, Omar Federico Ordoñez, Luciano José Martinez, María Eugenia Farías, Virginia Helena Albarracin

**Affiliations:** ^1^Laboratorio de Investigaciones Microbiológicas de Lagunas Andinas (LIMLA), Planta Piloto de Procesos Industriales y Microbiológicos (PROIMI), CCT, CONICET, Av. Belgrano y Pasaje Caseros, 4000 San Miguel de Tucumán, Argentina; ^2^Centro de Investigaciones y Servicios de Microscopía Electrónica (CISME), CCT-CONICET Tucumán-Universidad Nacional de Tucumán, Finca El Manantial, Camino de Sirga, 4107 Yerba Buena, Tucumán, Argentina

## Abstract

High levels of arsenic present in the High Altitude Andean Lakes (HAALs) ecosystems selected arsenic-resistant microbial communities which are of novel interest to study adaptations mechanisms potentially useful in bioremediation processes. We herein performed a detailed characterization of the arsenic tolerance profiles and the biofilm production of two HAAL polyextremophiles,* Acinetobacter* sp. Ver3 (Ver3) and* Exiguobacterium* sp. S17 (S17). Cellular adherence over glass and polypropylene surfaces were evaluated together with the effect of increasing doses and oxidative states of arsenic over the quality and quantity of their biofilm production. The arsenic tolerance outcomes showed that HAAL strains could tolerate higher arsenic concentrations than phylogenetic related strains belonging to the German collection of microorganisms and cell cultures (Deutsche Sammlung von Mikroorganismen und Zellkulturen, DSMZ), which suggest adaptations of HAAL strains to their original environment. On the other hand, the crystal violet method (CV) and SEM analysis showed that Ver3 and S17 were able to attach to solid surfaces and to form the biofilm. The quantification of biofilms production in 48 hours' cultures through CV shows that Ver3 yielded higher production in the treatment without arsenic cultured on a glass support, while S17 yield higher biofilm production under intermediate arsenic concentration on glass supports. Polypropylene supports had negative effects on the biofilm production of Ver3 and S17. SEM analysis shows that the highest biofilm yields could be associated with a larger number of attached cells as well as the development of more complex 3D multicellular structures.

## 1. Introduction

Arsenic (As) is recognized as one of the world's health greatest environmental hazards [1]. Arsenic pollution occurs mainly by volcanic activity but also by means of human activities required for the processing of geological materials such as coal and metalliferous ores or due to the application of biocides by agricultural and forestry industries [2]. It is known that long-term exposure to arsenic produces a broad array of effects in human health defined as the HACRE disease (Chronic Endemic Regional Hydroarsenicism) [3]. HACRE is quite frequent in many countries in the world [1, 3–5]. In these regions, As is naturally concentrated in rocks and soils and chronically consumed by people using groundwater and stream water without any treatment [1]. HACRE causes particular kinds of cancer and noncancerous diseases with dermal, reproductive, pulmonary, and neurologic effects [3]. For this reason, the World Health Organization (WHO) recommends a limit of 10 *μ*g As/L in water for human's consumption [6]. Conversely, high levels of arsenic were measured in drinking water in Argentina with extreme values that exceed the safe drinking water limit recommended by World Health Organization as well as the Argentine drinking water standard of 50 *μ*g/L (above 1000 *μ*g/L). Moreover, 99% of the groundwater for human consumption of many populations of Argentina is above 10 *μ*g/L affecting at least ca. 4,000,000 people [3].

Thus, it is important to develop methods and technologies for efficient removal of arsenic from soil and water. Chemical methods include coagulation, filtration, lime softening, activated alumina adsorption, ion exchange, reverse osmosis, reversal electrodialysis, and nanofiltration [5]. Likewise, new technologies have implemented biological methods in a process called bioremediation with important applications for heavy-metals and metalloids decontamination processes [7]. Among bioremediation techniques, microbial catalysis is preferred as it is less time-consuming, requires no chemical dosing, and is environmentally friendly and potentially cheap [7].

The screening for microbes with essential genes coding for phenotypes responsible for metal-microbe interactions and biofilm formation are crucial steps for designing successful bioremediation strategies for metals and radionuclides [8, 9]. Many reports describe the importance of combining tolerant/resistant strains in biofilm arrays for bioremediation [10, 11], referring to the set of cells embedded in an exopolysaccharide (EPS) matrix as “biofilm” [12]. The extracellular polymeric substances secreted by the biofilm promote the immobilisation, sorption, sequestration, and precipitation of heavy metals/metalloids [10, 12]. On the other hand, microbial communities gathered in biofilms are more stable and promote long-term survival of colonies when exposed to hard environmental stresses, i.e., high metals concentrations or other toxic compounds. In addition, the disposition of cells on particles or surfaces facilitates the separation of the cells from products in solution and may also allow the use of continuous reactors while avoiding washout [13, 14].

High Altitude Andean Lakes (HAALs) are shallow lakes situated in northwest Argentina, Chile, and Bolivia in the so-called Puna-High Andes region, at altitudes higher than 3,000 m [15, 16]. Local geochemistry and active volcanism are responsible for the high concentration of arsenic in HAAL water, soil, and sediments that normally range between 800 *μ*g As/L and 33,800 *μ*g As/L [17, 18], with extreme abnormal values ranging from 115 to 234 mg/L in some lakes [19]. Such arsenic accumulation in combination with high salinity, arid landscape with scarce precipitation (147 mm annually), wide daily thermal amplitude (35°C), oligotrophy, alkali pH, and the worldwide greatest index of UV radiation make the HAAL exceptional novel sources of polyextremophiles with high biotechnological potential [15, 16, 20]. In previous works, we reported a high diversity of HAAL isolated strains with intrinsic resistance to arsenic, UV radiation, salinity, and antibiotics [16, 20]. Among them, we have identified* Acinetobacter* sp. Ver3 (Ver3) as a model strain for study of molecular resistance mechanisms involved in UV-resistance [21–24]. Also,* Exiguobacterium* sp. S17 (S17) was able to grow in synthetic media added with high arsenic content showing differential protein expression and the presence of the Acr3 gene involved in arsenic tolerance mechanisms [25–27]. In this follow-up work, we performed a detailed characterization of arsenic tolerance profiles and biofilm production of these two HAAL polyextremophiles. Cellular adherence over glass and polypropylene surfaces were evaluated together with the effect of increasing doses and oxidative states of arsenic over the quality and quantity of their biofilm production.

## 2. Material and Methods

### 2.1. Strains and Culture Conditions

*Acinetobacter* sp. Ver3 and* Exiguobacterium* sp. S17 belong to the HAALs strains collection of LIMLA-PROIMI-CONICET [16]. Ver3 was isolated from the surface water of L. Verde while S17 was isolated from the stromatolites of L. Socompa.* Exiguobacterium aurantiacum* (DSM 6208),* Acinetobacter johnsonii* (DSM 6963) and* Acinetobacter baumannii* (DSM 30007) were obtained from the DSMZ-German collections of microorganisms and used as controls. Axenic glycerol-freeze cultures were aerobically grown in Luria-Bertani (LB, Britania) diluted at 50% (LB50) and cultured at 30°C with moderate agitation (means 120 rpm in all cases). DSM-30007 was incubated at 37°C. The cultures were maintained in LB agar for further inoculations.

### 2.2. Arsenic Resistance Profiles

All cultures were grown to OD 600nm ≈ 0.6 (109 UFC per milliliter of culture) and then centrifuged at 5,000 rpm for 10 minutes (min). Pellets were washed twice with physiological solution (PS) and suspended again to OD 600nm ≈ 0.6 in PS. The cultures were sequentially diluted 1:10 six times and 5 *μ*l of each dilution was plated on LB50 agar supplemented with arsenic increasing concentrations: i.e., 50 mM, 100 mM, 150 mM, 200 mM, 250 mM, and 350 mM for arsenate (As [V]) (Na2HAsO4.7H2O, Biopack) and 2.5 mM, 5 mM, 7.5 mM, 10 mM, 12.5 mM, and 15 mM for arsenite (As [III]) (NaAsO2, Fluka analytical).* Acinetobacter baumannii* DSM 30007 was used as a sensitive control to check the toxicity of the arsenic solutions. DSM 30007 plates were incubated at 37°C, whereas HAAL strains were incubated at 30°C.

### 2.3. Growth Curves under Arsenic Supplementation

Glass flasks (glass bottles) (100 ml) with 50 ml of LB50 were inoculated to OD600nm ≈ 0.08 and incubated at 30°C with moderate agitation. The arsenic concentrations applied in this assay were As [V] 50 mM or As [III] 2.5 mM. Control cultures were run in LB50 without arsenic supplementation. The growth was measured every 3 hours (h) by taking 1 ml of culture and measuring OD600nm.

### 2.4. Quantification of Biofilms

Biofilms of Ver3 and S17 were produced in LB50 media with or without arsenic supplementations at different concentrations: As [V] 50 mM, 150 mM, 250 mM, and 350 mM and As [III] 2.5 mM, 7.5 mM, and 12.5 mM, respectively. Overnight cultures were centrifuged and washed twice with PS. Aliquots of 20 ml of a fresh inoculum at OD600nm ≈ 0.08 were added into glass and polypropylene flasks and incubated for 48 h at 30°C with moderate agitation. After measuring the OD600nm, the supernatants were discarded and containers were rinsed with running tap water three times and allowed to dry upside down in stove at 60°C. The cells attached to flasks surfaces were visualized and quantified by staining each container with 20 ml of crystal violet 1% (CV), for 3 hours. The excess of CV was discarded and containers were washed with tap water and let dry upside down in stove at 60°C. The binding CV was solubilized with ethanol 96.8% in shaker for 16 h and the resulting solution was read at OD 570nm to be used as an indirect estimator of the amount of cellular adherence [28, 29]. A detailed protocol is loaded in the Method detail section of the supplementary file. In this assay, OD was measured with Elisa Reader spectrophotometer (Multiskan Go, Thermoscientific). Duplicate cultures were performed in parallel and supervised macroscopically along 10 days.

### 2.5. Biofilms Imaging with Scanning Electron Microscopy

Microscopy glass dishes and bits of polypropylene were glued with nail polish into the inner surface of glass flasks to provide a surface for the adhesion of the samples. Flasks were autoclaved for 15 minutes at 120°C and let dry. Aliquots of 20 ml of Ver3 or S17 fresh inoculum at OD 600nm ≈ 0.08 were added into the flasks and supplemented with/without arsenic concentration of As [V] 50 mM, 150 mM, and 350 mM or As [III] 2.5 mM and 7.5 mM. Cultures were incubated for 48 h with moderate agitation. After incubation, the biofilms samples were carefully detached and gently rinsed with PS and fixed with Karnovsky fixative solution for 48 h. The samples were dehydrated successively with alcohol 30%, 50%, 70%, 90%, and 100% for 10 min each and, finally, maintained in acetone 24h and then complete dehydration was carried out with the critical point technique. Samples were mounted on scanning electron microscopy sampler stubs and gold coated. The procedure was carried out in Centro de Investigaciones y Servicios de Microscopía Electrónica (CISME). A total of 300 images were analysed.

### 2.6. Cell Clumping Analysis

Cellular adhesions on SEM pictures under the influence of arsenic were described by measuring two parameters in at least four SEM microphotographs at 5000X for each treatment. Those parameters are the total number of visible cells and the number of nonaggregate cells present in the sample. Because Ver3 and S17 are predominately diplococci in shape, our criteria to determine if linked cells are or not a cellular aggregate, more than three cells (six cocci) have to be closely linked (to exclude the dividing pairs of cells) [30], and each individual diplococci cell was considered and counted as two cells. The degree of cellular clumping was estimated as the probability that a randomly chosen cell belongs to a cellular aggregate (see (1)), at the time of the fixation procedure and according to our criteria.

(1) $$\begin{array}{c} P=N\text{–}\frac{I}{N} \end{array}$$

Equation (1): the probability (P) that a randomly chosen cell belongs to a cellular aggregate was calculated based on the number of nonforming aggregate cells (I) and the total number of cells (N) present in the sample at the time of the fixation procedure. The pictures chosen to be present in this work were those that obtained scores closest to the average of each treatment.

### 2.7. Statistical Analysis

Statistical analysis was carried out using R statistical software [31]. The simultaneous analysis of the effect of arsenic doses levels and the types of supports and their interactions were analysed through general linear models. Compact letter displays were generated by “agricolae” package using Tukey HSD test. Data analysis was based on at least three biological repetitions.

## 3. Results

### 3.1. Arsenic Resistance Profiles of HAAL's Strains

**Figure 1:**
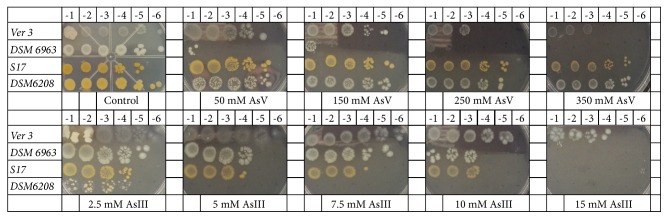
Arsenic tolerance profiles through agar drop plate method. Serial dilutions (top) of HAAL an DMSZ strains (left) were plated on LB50 amended with increasing concentrations of As [V] and As [III] (base of the figures), respectively.

The arsenic resistance profiles of the five strains were tested by plating serial dilutions on LB50 agar containing increasing concentrations of either As [V] or As [III] as sodium salts. As a general fact, HAAL's indigenous strains were much more resistant than the phylogenetically related DSM strains (Figure 1). Moreover,* A*.* baumannii *DSM 30007 could not grow in any of the arsenic concentrations tested (data not shown).

In LB50 agar supplemented with As [V] 50-250 mM, the viability of Ver3 was not significantly affected while it remains viable even at concentrations of 350 mM. In contrast,* A*.* johnsonii* DSM 6963 presented a significant inhibition in growth in the range of As [V] 50-250 mM while no growth was recorded at 350 mM. Ver3 was able to grow at all arsenite concentrations tested (up to As [III] 15 mM). In contrast, DSM 6963 presents moderate growth at As [III] 10 mM but was completely inhibited at 15 mM (Figure 1).

The tolerance response of S17 was tested and compared with* E*.* aurantiacum* DSM 6208. Under the conditions used in this study, S17 and DSM 6208 showed similar As [V] tolerance profiles, being able to grow at all tested concentrations. In contrast, S17 was able to grow in media containing up to 10 mM As [III] while DSM 6208 was completely inhibited at 5 mM (Figure 1).

### 3.2. Growth Course under Arsenic-Stress

**Figure 2:**
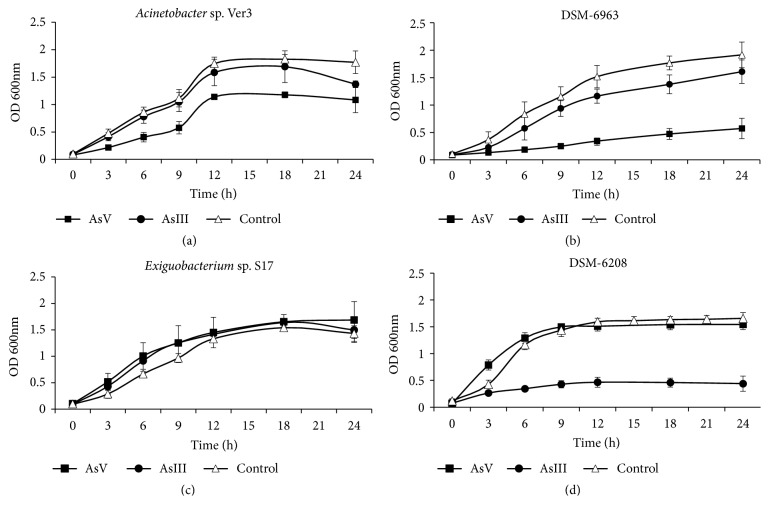
Growth curves of HAAL and DSMZ strains grown with/without 50 mM As [V] or 2.5 mM As [III]. (a)* Acinetobacter* sp. Ver3, (b)* Acinetobacter johnsonii* DSM 6963, (c)* Exiguobacterium* sp. S17, and (d)* Exiguobacterium aurantiacum* DSM 6208.

The tolerance profiles of HAAL and DSMZ strains were also tested in liquid media LB50 supplemented with As [V] 50 mM or As [III] 2.5 mM (Figure 2, Table S1).

In liquid media, it was also evident that HAAL strains better tolerated the addition of arsenic salts (As [III] and As [V]) than DSMZ strains. Nevertheless, it should be noted that there is a common profile for* Acinetobacter* strains, which are almost not affected by the addition of As [III] while resulting as clearly inhibited under As [V] stress. In turn, DSM 6208 growth was strongly diminished under As [III] addition while it was not affected by As [V] when compared to the control treatment (Figure 2).

In nonamended LB50, Ver3 reached a final OD600nm of ~1.8 with a duplication rate of ~30% per hour (in exponential phase). A similar profile was observed in media amended with As [III] 2.5 mM. However, the addition of As [V] 50 mM reduces the growth rate by ~10% per hour, reaching a final OD600nm that is 30% lower than in the control treatment. Ver3 and DSM 6963 have both similar growth rates and final yields in treatments with 2.5 mM As [III] or in control conditions. In media amended with 50 mM As [V], DSM 6963 growth rate diminished, which produced a displacement of the exponential phase influencing its final yield: a 60% lower than in the control treatment and 50% of Ver3 yield under the same culture conditions (Table S1; Figures 2(a) and 2(b)).

S17 grew in media without arsenic as well as in medium containing either As [V] 50 mM or As [III] 2.5 mM at similar growth rates (~30% per hour), reaching equivalent final yields (~1.5) in the three treatments. In turn, DSM 6208 growth rate was not affected by As [V]-supplementation, while in presence of As [III] 2.5 mM its growth rate was reduced to 16% per hour, with its final yield being three times smaller than the control (Table S1; Figures 2(c) and 2(d)).

### 3.3. Cellular Adherence and Biofilm Formation

Adherence of microbial cells to a support is the first step in biofilm formation. Thus, cellular adherence of HAAL's strains was studied under different arsenic treatments and on different supports (glass or polypropylene). Ver3 and S17 adherence to flasks surfaces was assessed macroscopically and quantified at 48 h of incubation by solubilizing the crystal violet attached to the cells and reading the OD570nm [28, 29].

Ver3 cultures were turbidly producing a white slime (probably due to exopolysaccharides, EPS) observed as mucus material in the culture medium, which after 48 h developed in dense biofilms attached to the wall or bottom surfaces (Figure S1). When cells were cultured for longer periods (144 h) (see Section 2.4), the biofilms appeared much denser in cultures containing 50 mM As [V] and As [III] 2.5 mM (Figures S1 b and c). However, these biofilms were not considered in the quantification analysis since its formation may be due to other stress factors (nutrient depletion, wastes, or secondary metabolites) rather than to arsenic or the support.

**Figure 3:**
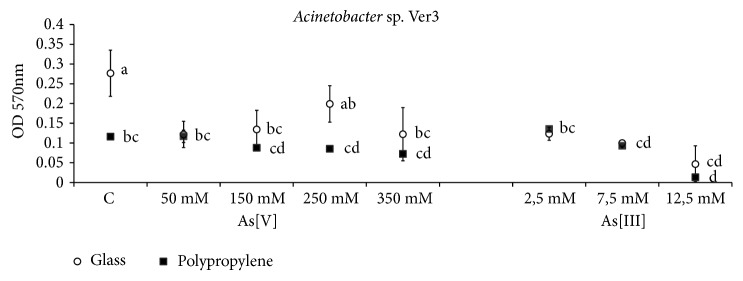
Cell adhesion of* Acinetobacter* sp. Ver3 on glass and polypropylene surfaces. Quantification is based on crystal violet method. Compact letters display is based on HSD Tukey test.

The quantification of Ver3 cell adherence in 48 h cultures (Figure 3) shows that the adhesion to solid surfaces was significantly greater over glass in cultures without arsenic addition (p <0.05), while the supplementation of arsenic or the presence of polypropylene surfaces had retardant or inhibitory effects on the development of the biofilm. Therefore, the statistical analysis shows that the arsenic doses (p < 0.001), the type of support (p < 0.001), and the interaction between the two factors (p < 0.001) had significant negative effects on the production of biofilms by Ver3.

**Figure 4:**
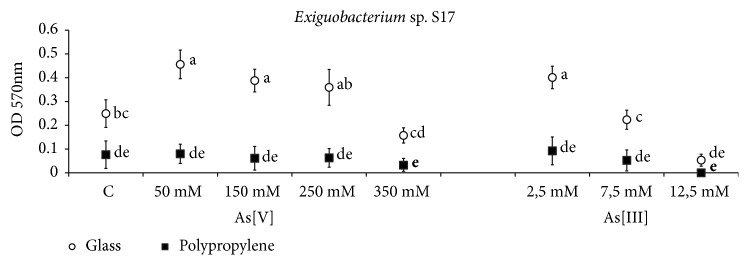
Cell adhesion of* Exiguobacterium *sp. S17 on glass and polypropylene surfaces. Quantification is based on crystal violet method. Compact letters display is based on HSD Tukey test.

On the other hand, S17 was a strong producer of a mature biofilm at 48 h of incubation, developing thick "rings" of EPS at the air-liquid interface and also releasing them to the culture medium. Likewise, at 48 h of incubation, it is possible to notice that both arsenic supplementation and the type of support have determinant effects on the production of biofilms by S17. As shown in Figure S2, arsenic exposure induces the development of biofilm (Figure S2 a) while polypropylene surfaces avoid cell adherence (Figure S2 b). Thus, the statistical analysis shows that cultures in glass flasks treated with As [V] 50-250 mM and As [III] 2.5 mM had the highest cellular adhesion patterns while it was retarded under control conditions and with As [V] 350 mM or As [III] 7.5 mM and was completely inhibited on polypropylene surfaces (Figure 4).

### 3.4. Cell Clumping Analysis by SEM Imaging

The formation of biofilms after 48 h was imaged using scanning electron microscopy to determine the cell adherence and clumping mechanisms in much detail. The measurement takes into account the total number of visible cells per sample (N) (from each SEM picture) and the probability that a randomly chosen cell belongs to a cellular aggregate (P) (see (1)). We chose the area for quantification below the EPS “rings” since, inside it, all samples were very similar, but outside of its boundaries the cellular aggregation is smaller and makes the qualitative as well as quantitative comparative analysis possible (Figure 6).

**Figure 5:**
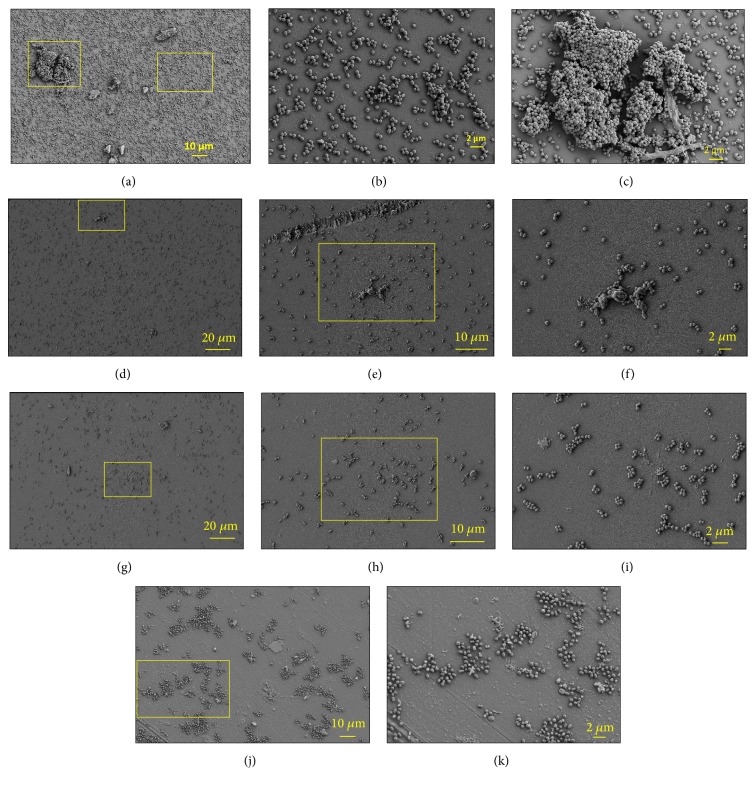
SEM “set-in” microphotographs of* Acinetobacter *sp. Ver3 cell adhesion on glass (a–i) and polypropylene (j-k) surfaces. (a–c) Control treatment, (d–f) As [III] 2.5 mM treatment in representation of As [V] 50-150 mM treatments, and As [III] 2.5 mM proper. (g–i) As [III] 7.5 mM treatment in representation of As [V] 350 mM and As [III] 7.5 mM proper. (j-k) Cellular adhesion under control conditions on polypropylene surfaces in representation of any arsenic concentration since there were no significant differences between treatments. Yellow boxes are the magnification areas.

SEM analysis shows that Ver3 adhered to both types of surfaces, but there were differences in the distribution, density, and cell aggregations patterns. On glass surfaces under control conditions (Figures 5(a), 5(b), and 5(c)), the cells were uniformly distributed forming microcolonies of 10-30 cells (Figure 5(b)) that eventually fuse to develop multicellular three-dimensional (3D) conglomerates (Figure 5(c)). Under these conditions, the individual cells constitute a lower proportion of the total cells per sample.

The addition of arsenic supplements in Ver3 cultures caused the reduction in the number of adhered cells per sample and affects the formation of microcolonies and 3D structures (Figures 5(d)–5(i)). Also, the types of surface material caused clear differences on the adhesion pattern (Figures 5(j) and 5(k)).

Ver3 cultures grown on glass surfaces and treated with As [V] 50-150 mM or As [III] 2.5 mM show similar adhesion behaviour; we observed low densities of cell aggregates and high abundance of individual cells (Figures 5(d)–5(f)). However, at higher arsenic concentrations such as As [V] 350 mM and As [III] 7.5 mM, cells were grown predominantly as small aggregates of 6-15 cells only (Figures 5(g)–5(i)), maybe a product of the cellular division of resistant phenotypes. In contrast, cultures grown on polypropylene surfaces at any arsenic concentrations show the same adhesion pattern with no significant differences on the number of attached cells per sample and with cells distributed in monolayer patches without cell-cell contact and interspersed with areas devoid of cells (Figures 5(j) and 5(k)).

**Figure 6:**
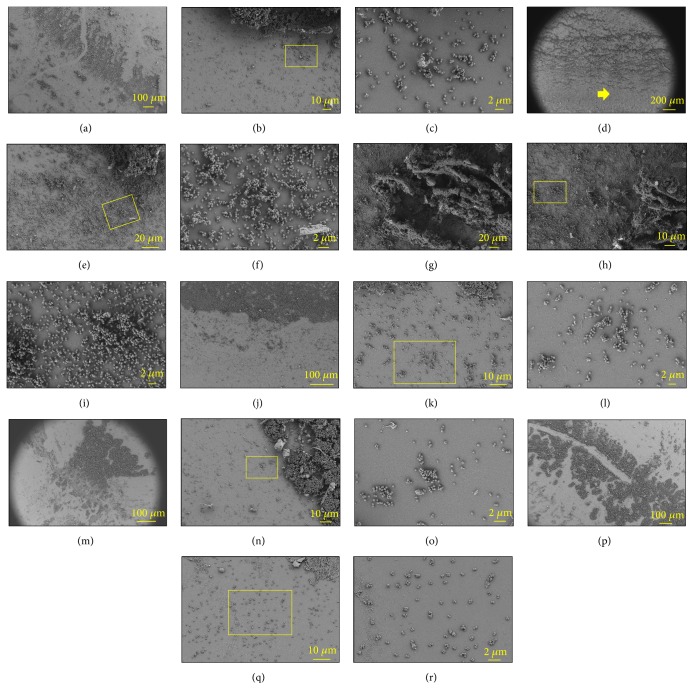
SEM “set-in” microphotographs of* Exiguobacterium* sp. S17 cell adhesion on glass surface. Control (a–c), As [V] 50 mM (d–f), As [V] 150 mM (g–i), As [V] 350 mM (j–l), As [III] 2.5 mM (m–o), and As [III] 7.5 mM (p–r). Yellow boxes are the magnification areas.

**Figure 7:**
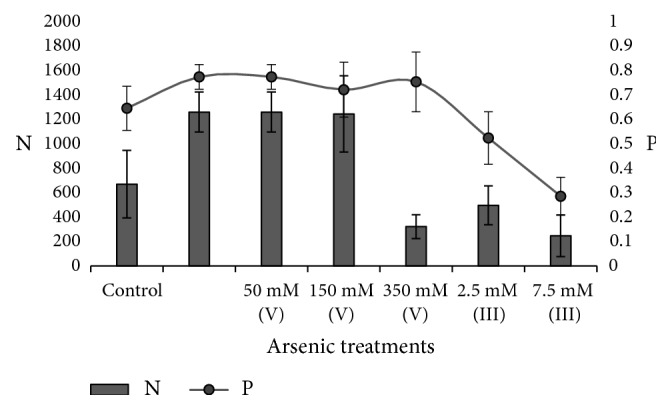
Cellular adherence and cell clumping analysis of* Exiguobacterium* sp. S17. Bars represent the number of visible cells present in the sample (N) and the line represents the degree of cellular clumping as the probability that a randomly chosen cell belongs to a cellular aggregate (P) at the time of the fixation procedure. In the horizontal axe the arsenate and arsenite increasing concentration.

S17 strongly adheres to glass surfaces; however no adherence was detected on polypropylene surfaces by SEM images in 48 h cultures. Likewise, arsenic had strong effects on cell adhesion directly influencing the number of attached cells per sample and the probability of cell-cell aggregation and, therefore, on the biofilm structure (Figures 6 and 7).

Figure 6 shows the adhesion pattern of S17 below the EPS rings—indicated by a yellow arrow in Figure 6(d). Arsenic had a clear effect on the 3D structure of the rings and also on the consistency of its boundaries; cultures treated under control conditions, or upon addition of As [V] 350 mM or As [III] 2.5-7.5 mM, showed hard rings boundaries with abrupt changes on the cell aggregation outside the ring (Figures 6(a), 6(j), 6(m), and 6(p)). In contrast, cultures treated with As [V] 50-150 mM displayed rings with diffuse edges and conspicuous aggregation patterns to long extent outside the ring (Figure 6(d)). Thus, the highest amounts of adhered cell per sample (1260 ± 164 and 1250 ± 320) with high cellular aggregation probabilities (0.77 and 0.72) were found in treatments containing As [V] 50-150 mM, respectively (Figure 7), in which the microcolonies grow in width and height to form large complex 3D multicellular structures both inside the ring and outside it (Figures 6(d)–6(i)).

In the control cultures, the rings were constituted by many layers of cells although they acquire flattened appearance with clearly delimited edges. Outside the ring, the number of adhered cells per sample was reduced (650 ± 300) in comparison with treatments containing As [V] 50-150 mM. Also, cells developed small aggregates of 10-40 cells and the proportions of aggregated-nonaggregated cells were approximately equivalent; therefore P reached 0.60 (Figures 6(a)–6(c) and 7).

Adhesions under As [V] 350 mM or As [III] 2.5-7.5 mM had the lowest amount of attached cells per sample (Figure 7) and were structurally less complex (Figures 6(j)–6(r)). Under As [V] 350 mM, the rings were flattened with delimited edges and constituted by two to three stacked layers of cells and, outside of it, the few cells (320 ± 100) were predominantly aggregated (P = 0.75) (Figures 6(j)–6(l)). Similarly, under As [III] 2.5-7.5 mM, the rings were clearly delimited (Figures 6(m) and 6(p)), but while under As [III] 2.5 mM an equal proportion of aggregated-nonaggregated cells were observed (P = 0.50); under As [III] 7.5 mM the amount of adhered cells was scarce and predominantly not aggregated or forming groups of 4-6 cocci (P = 0.30) (Figures 6(o), 6(r), and 7).

## 4. Discussion

Organisms respond to their environment adapting to their variables and in many cases, modifying their biology and development. Therefore, extreme environments or contaminated sites are “promised lands” for searching novel resistant organisms with biological mechanisms of potential biotechnological use [9, 32]. That is the case for HAAL's ecosystems due to their particular array of extreme conditions [15, 16, 20]. The study of these complex communities and their adaptive mechanisms is of full importance to understand how life under harsh conditions occurs and, in turn, they can provide the raw material for the development of biotechnological processes.

Our results showed the tolerance profile to As [V] and As [III] of two model polyextremophilic strains isolated from HAAL:* Acinetobacter* sp. Ver3 and* Exiguobacterium* sp. S17 compared to their phylogenetically related strains,* Acinetobacter johnsonii* (DSM 6963),* Acinetobacter baumannii* (DSM 30007) and* Exiguobacterium aurantiacum* (DSM 6208). Interestingly, the arsenic oxidative states affected each genus differentially. While* Acinetobacter* spp. growth was negatively affected by As [V], strains belonging to* Exiguobacterium* grew better or were not affected under similar conditions. Anderson and Cook [33] conducted a comparable study on* Exiguobacterium* sp. WK6 and explain this phenomenon as result of a detoxifying arsenate to arsenite process in which the external pH becomes alkaline, allowing the bacteria to grow for extended periods of time. As discussed by Ordoñez et al., this fact should be explained by the promotion of a more active metabolism as suggested by the overexpression of glycolysis proteins in presence of As [V] [25, 27].

Also, our results show that HAAL strains were more resistant to arsenic than DSMZ strains, being able to grow at the higher concentration of both arsenic oxidative states. This could be explained by the fact that DSMZ strains were isolated from nonarsenic environments [34, 35], suggesting that the tolerance profiles are in agreement to the original environment. Similar patterns were found in our laboratory when HAAL strains were exposed to arsenic [27, 36] or tested under artificial UV radiation [21–24], supporting our hypothesis.

Microbial biofilms can be defined as sessile microbial consortia developing in a three-dimensional structure; these multicellular communities containing prokaryotic or/and eukaryotic cells are embedded in a matrix produced partially or completely by the microbial community [12]. Biofilm formation is a multistage process in which the first step is the microbial adhesion. In a subsequent process, the cells produce and accumulate an extracellular matrix composed of one or more polymeric substances such as proteins, polysaccharides, humic substances, extracellular DNA, and signaling molecules for communication [10]. Thus, to study microbial biofilms in HAAL strains we study both processes, i.e., adhesion and aggregation.

S17 and Ver3 varied in their affinity to attach on glass or polypropylene. Cell-surface interaction includes Lifshitz-van der Waals, electrostatic, and hydrophobic forces [37]. Nevertheless, bacterial cell surface and substratum surface hydrophilicity could play a more important role in the adherence process [38]. SEM analysis shows that* Acinetobacter* sp. Ver3 adhered to both types of surfaces while* Exiguobacterium* sp. S17 only adheres to glass surfaces (Figures 5 and 6). We suggest that these differences in strain-surface affinities are associated with the type and properties of the EPS secreted by each strain, as EPS are recognized to be the primary structures in early stage attachment [10, 12, 39] and because we failed to detect cellular pilis and other cell structures mediated the early stage cell-surface contact such as DNA and proteins.

Arsenic-affected cells adherence by HAAL strains. This effect cannot be explained as a cause of an arsenic electrostatic phenomenon since the highest biofilm production was found without arsenic (Ver3) or at intermediate concentrations of arsenic (S17) (Figures 3 and 4). Therefore, the effect of arsenic on diminishing or promoting adhesion must be through modification of the cell membranes.

The addition of arsenic at intermediate concentrations on S17 cultures caused the appearance of macroscopic biofilm structures, both, dispersed in the medium and stuck at the air-liquid interface of the flasks (Figure S2). SEM showed that the biofilm developed at the air-liquid interface varied in complexity depending on the arsenic doses (Figure 6). The air-liquid interface is a valuated niche for microorganism since it accumulates nutrients, and, there, the oxygen concentration is higher than on the bulk medium but, presumably, only the second may occur in shaking experimental rich medium cultures [12]. Therefore, there are two reasons that could explain the fact that* Exiguobacterium* sp. S17 produced more complex tick EPS rings with continuous edges in cultures amended with As [V] 50-150 mM (Figure 6), the ratio oxygen/arsenic and the proliferation of resistant phenotypes. In control cultures, oxygen is equally distributed in the bulk medium, so cells can thrive both in the bulk medium as planktonic or attached at the air-liquid interface; therefore, there is low competition for the air-liquid interface niche and the EPS rings have a low complexity. However, in As [V] 50-150 mM amended cultures, oxygen and arsenic compete in cells respiratory chain, so the toxicity of the medium will be lower and will enable cell population survival where the oxygen/arsenic ratio is higher, at the air-liquid interface. So, at intermediate arsenic concentrations, there is high demand for oxygen and therefore many cells will compete for the air-liquid interface niche generating rings of high thickness and complexity (Figures 6(d)–6(i), Figures S1 and S2). In contrast, under higher toxic conditions such as the cultures amended with As [V] 350 mM and As [III] 7.5 mM, we suggest that only the small proportion of the very resistant phenotypes that could survive will be able to stick on the support surface leading to the development of flat and low complexity rings and, outside it, only small microcolonies could be formed as the product of the division of resistant phenotypes that remain as “persisters” (Figure 6(r)) [40–42]. Ver3 shows the same pattern under high arsenic doses (Figure 5(i)).

## 5. Concluding Remarks

The results presented in this work contribute to the understanding of early stage of the biofilm formation by HAAL strains and the influence of arsenic on promoting cellular aggregation. Likewise, these results highlight HAAL's ecosystem as a source of novel bacterial species of biotechnological potential, i.e., arsenic bioremediation.

This work is an important contribution to the knowledge of the biology and ecology of microbial extremophiles from High-Altitude Andean Lakes, specifically contributing to elucidating the biofilm as a mechanism to resist polyextreme conditions. Moreover, this work increases patrimonial and microbiological value of Andean ecosystems and in this way supports the conservation and protection of these landscapes from uncontrolled human activities such as the ongoing mining and adventure tourism projects.

## Data Availability

The data corresponding to growth courses, biofilm production, SEM methodology and cell clumping analysis, as well as the imaging data used to support the findings of this study are included within the supplementary information file.

## Supplementary Materials

Supplementary Materials This file is divided into three sections containing the supplementary figures, the data sets, and a detailed protocol that we used to acquire SEM images. (1) Supplementary Figures: in this section we provide the supplementary figures mentioned in the main manuscript. Table S1 shows the means growth rates with their respective standard deviation of* Acinetobacter* sp. Ver3 (Ver3),* Acinetobacter johnsonii* (DSM 6963),* Exiguobacterium* sp. S7 (S17), and* Exiguobacterium aurantiacum* (DSM 6208) grown with or without 50 mM As [V] or 2.5 mM As [III], respectively. Figures S1 and S2 show the macroscopic formation of biofilm over the support surfaces of* Acinetobacter* sp. Ver3 and* Exiguobacterium* sp. S17, respectively (see Section 3.3 of the main manuscript). (2) Supplementary Data Files: the data corresponding to growth curves, biofilm production, and cell clumping analysis as well as imaging data used to support the findings of the study are provided in this section.
