# Supplementary material for: Polyextremophilic Bacteria from High Altitude Andean Lakes: Arsenic Resistance Profiles and Biofilm Production
Source: Biomed Res Int. 2019 Feb 21;2019:1231975. doi: 10.1155/2019/1231975 (PMC6409018; doi:10.1155/2019/1231975)
Supplement: Supplementary Materials — This file is divided into three sections containing the supplementary figures, the data sets, and a detailed protocol that we used to acquire SEM images. (1) Supplementary Figures: in this section we provide the supplementary figures mentioned in the main manuscript. Table S1 shows the means growth rates with their respective standard deviation of Acinetobacter sp. Ver3 (Ver3), Acinetobacter johnsonii (DSM 6963), Exiguobacterium sp. S7 (S17), and Exiguobacterium aurantiacum (DSM 6208) grown with or without 50 mM As [V] or 2.5 mM As [III], respectively. Figures S1 and S2 show the macroscopic formation of biofilm over the support surfaces of Acinetobacter sp. Ver3 and Exiguobacterium sp. S17, respectively (see Section 3.3 of the main manuscript). (2) Supplementary Data Files: the data corresponding to growth curves, biofilm production, and cell clumping analysis as well as imaging data used to support the findings of the study are provided in this section. [file 1231975.f1.pdf]

Supplementary Materials File

This file is divided into three sections containing the supplementary figures, the data sets, and a detailed protocol that we used to acquire SEM images.

1. Supplementary Figures

In this section we provide the supplementary figures mentioned in the main manuscript. Table S1 shows the means growth rates with its respective standard deviation of *Acinetobacter* sp. Ver3 (Ver3), *Acinetobacter Johnsonnii* (DSM 6963), *Exiguobacterium* sp. S7 (S17) and *Exiguobacterium aurantiacum* (DSM 6208) grown with or without 50 mM As [V] or 2.5 mM As [III], respectively.

Figures S1 and S2 shows the macroscopic formation of biofilm over the support surfaces of *Acinetobacter sp.* Ver3 and *Exiguobacterium* sp. S17, respectively (see section 3.3 of the main manuscript).

Treatment	Ver3	DSM 6963	S17	DSM 6208
Control	29.71 ± 3.74	32.12 ± 5.35	30.57 ± 3.04	35.10 ± 8.75
AsV 50 mM	19.59 ± 4.57	16.62 ± 9.74	31.90 ± 6.44	29.15 ± 4.00
AsIII 2,5 mM	29.57 ± 4.42	31.18 ± 8.54	29.87 ± 9.02	16.65 ± 3.84

Table S1 - Growth rates (% per hour) of HAAL and DSMZ strains grown with/without 50 mM As [V] or 2.5 mM As [III].

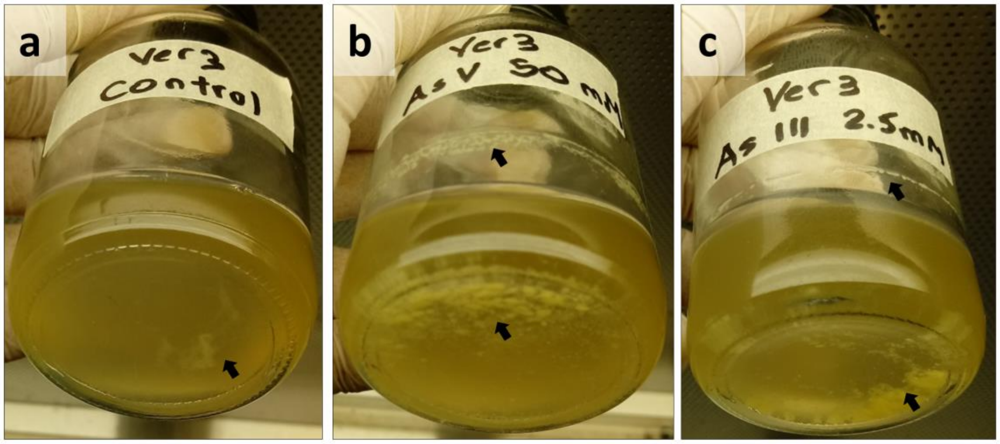

Figure S1 – Visual assessment of biofilm production by *Acinetobacter* sp. Ver3 in duplicate cultures (144 h), under a) Control, b) As [V] 50 mM and c) As [III] 2.5 mM treatments respectively, at six days of incubation. Black arrows indicate macroscopic biofilm structures.

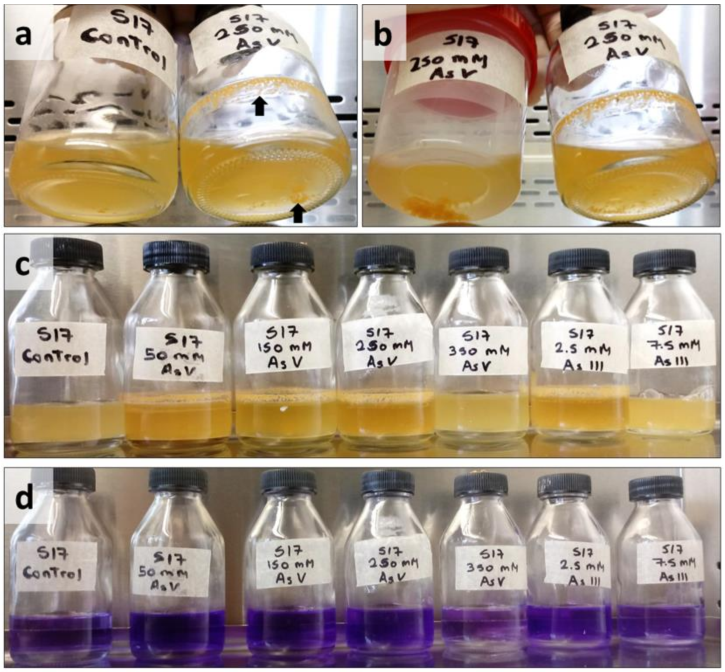

Figure S2 – Visual assessment of biofilm production by *Exiguobacterium* sp. S17 in 48 hours cultures, showing (a) the effect of arsenic on biofilm development in a representative comparison between the control and As [V] 250 mM treatments (black arrows shows macroscopic structures at the air-liquid interface and floating at the bottom of the flasks) and, (b) the effect of glass and polypropylene surfaces on cell–surface adhesion. Complete series of biofilm production on glass flask under arsenic increasing concentration c) before and (d) after staining with the crystal violet.

2. Supplementary Data Files

The data corresponding to either, growth curves, biofilm production and cell clumping analysis, as well imaging data used to support the findings of the study are provided in this section.

2.1. Growth Curves Data

You should know the following information about the Dynamic data table of growth curves.

1- The first line consists of labels.
2- The first column contains strain names; *Acinetobacter* sp. Ver3 (Ver3), *Acinetobacter johnsonii* (DSM 6369), *Exiguobacterium* sp. S17 (S17) and *Exiguobacterium aurantiacum* (DSM 6208).
3- The second column contains the sampling time in hours.

4- The third column contains the arsenic treatment applied to the cultures; “As V” correspond to arsenate, “As III” to arsenite and, “C” for control treatment. Consider that the arsenic concentrations used in this assay were As V 50 mM and AsIII 2.5 mM.
5- The fourth column contains the growth measurements based on the optical density (DO) read at 600 nm.
6- The table to the left of the reading frame corresponds to the continuation of the table on the right.

Strain	Time	Treatment	DO 600 nm
Ver3	0	As V	0.08
Ver3	0	As V	0.08
Ver3	0	As V	0.08
Ver3	0	As V	0.08
Ver3	0	As V	0.08
Ver3	0	As V	0.08
Ver3	3	As V	0.27
Ver3	3	As V	0.25
Ver3	3	As V	0.23
Ver3	3	As V	0.23
Ver3	3	As V	0.14
Ver3	3	As V	0.17
Ver3	6	As V	0.5
Ver3	6	As V	0.5
Ver3	6	As V	0.41
Ver3	6	As V	0.37
Ver3	6	As V	0.28
Ver3	6	As V	0.37
Ver3	9	As V	0.69
Ver3	9	As V	0.7
Ver3	9	As V	0.62
Ver3	9	As V	0.51
Ver3	9	As V	0.41
Ver3	9	As V	0.53
Ver3	18	As V	1.23
Ver3	18	As V	1.12
Ver3	18	As V	1.18
Ver3	24	As V	1.26
Ver3	24	As V	1.18
Ver3	24	As V	1.3
Ver3	0	As III	0.1

continue…			
DSM 6963	0	C	0.16
DSM 6963	0	C	0.17
DSM 6963	0	C	0.07
DSM 6963	0	C	0.06
DSM 6963	0	C	0.07
DSM 6963	3	C	0.52
DSM 6963	3	C	0.52
DSM 6963	3	C	0.24
DSM 6963	3	C	0.33
DSM 6963	3	C	0.23
DSM 6963	6	C	1.09
DSM 6963	6	C	1.02
DSM 6963	6	C	0.61
DSM 6963	6	C	0.84
DSM 6963	6	C	0.62
DSM 6963	9	C	1.41
DSM 6963	9	C	1.23
DSM 6963	9	C	0.97
DSM 6963	9	C	1.15
DSM 6963	9	C	1.01
DSM 6963	12	C	1.3
DSM 6963	12	C	1.7
DSM 6963	12	C	1.55
DSM 6963	18	C	1.9
DSM 6963	18	C	1.75
DSM 6963	18	C	1.65
DSM 6963	24	C	1.99
DSM 6963	24	C	2.1
DSM 6963	24	C	1.65
S17	0	As V	0.11
S17	0	As V	0.12

Ver3	0	As III	0.11
Ver3	0	As III	0.1
Ver3	0	As III	0.07
Ver3	0	As III	0.07
Ver3	3	As III	0.36
Ver3	3	As III	0.34
Ver3	3	As III	0.52
Ver3	3	As III	0.45
Ver3	3	As III	0.39
Ver3	6	As III	0.68
Ver3	6	As III	0.64
Ver3	6	As III	0.93
Ver3	6	As III	0.86
Ver3	6	As III	0.78
Ver3	9	As III	0.97
Ver3	9	As III	0.87
Ver3	9	As III	1.32
Ver3	9	As III	1.1
Ver3	9	As III	0.97
Ver3	12	As III	1.79
Ver3	12	As III	1.32
Ver3	12	As III	1.64
Ver3	18	As III	2
Ver3	18	As III	1.43
Ver3	18	As III	1.64
Ver3	24	As III	1.4
Ver3	24	As III	1.3
Ver3	24	As III	1.41
Ver3	0	C	0.12
Ver3	0	C	0.12
Ver3	0	C	0.11
Ver3	0	C	0.1
Ver3	0	C	0.07
Ver3	0	C	0.07
Ver3	3	C	0.41
Ver3	3	C	0.42
Ver3	3	C	0.57
Ver3	3	C	0.56
Ver3	3	C	0.49
Ver3	3	C	0.4
Ver3	6	C	0.79

S17	0	As V	0.08
S17	3	As V	0.7
S17	3	As V	0.43
S17	3	As V	0.42
S17	6	As V	1.18
S17	6	As V	0.72
S17	6	As V	1.12
S17	9	As V	1.21
S17	9	As V	1.3
S17	9	As V	1.25
S17	12	As V	1.71
S17	12	As V	1.14
S17	12	As V	1.5
S17	18	As V	1.71
S17	18	As V	1.5
S17	18	As V	1.75
S17	24	As V	1.75
S17	24	As V	1.31
S17	24	As V	2
S17	0	As III	0.11
S17	0	As III	0.08
S17	0	As III	0.08
S17	3	As III	0.6
S17	3	As III	0.34
S17	3	As III	0.35
S17	6	As III	0.91
S17	6	As III	0.92
S17	6	As III	0.91
S17	9	As III	1.21
S17	9	As III	1.3
S17	9	As III	1.25
S17	12	As III	−
S17	12	As III	−
S17	12	As III	−
S17	18	As III	1.55
S17	18	As III	1.7
S17	18	As III	1.66
S17	24	As III	1.3
S17	24	As III	1.76
S17	24	As III	1.43
S17	0	C	0.08

Ver3	6	C	0.81
Ver3	6	C	0.97
Ver3	6	C	0.98
Ver3	6	C	0.85
Ver3	6	C	0.76
Ver3	9	C	1.05
Ver3	9	C	1.05
Ver3	9	C	1.2
Ver3	9	C	1.38
Ver3	9	C	1.09
Ver3	9	C	0.92
Ver3	12	C	1.77
Ver3	12	C	1.85
Ver3	12	C	1.62
Ver3	18	C	1.85
Ver3	18	C	1.9
Ver3	18	C	1.73
Ver3	24	C	1.9
Ver3	24	C	2
Ver3	24	C	1.8
Ver3	24	C	1.68
Ver3	24	C	1.47
DSM 6963	0	As V	0.18
DSM 6963	0	As V	0.15
DSM 6963	0	As V	0.07
DSM 6963	0	As V	0.05
DSM 6963	0	As V	0.07
DSM 6963	0	As V	0.05
DSM 6963	0	As V	0.05
DSM 6963	3	As V	0.21
DSM 6963	3	As V	0.18
DSM 6963	3	As V	0.13
DSM 6963	3	As V	0.11
DSM 6963	3	As V	0.09
DSM 6963	3	As V	0.09
DSM 6963	3	As V	0.12

S17	0	C	0.13
S17	0	C	0.07
S17	0	C	0.08
S17	3	C	0.31
S17	3	C	0.39
S17	3	C	0.23
S17	3	C	0.21
S17	6	C	0.63
S17	6	C	0.74
S17	6	C	0.66
S17	6	C	0.63
S17	9	C	0.95
S17	9	C	1.08
S17	9	C	0.96
S17	9	C	0.87
S17	12	C	1.31
S17	12	C	1.38
S17	12	C	1.3
S17	18	C	1.48
S17	18	C	1.54
S17	18	C	1.6
S17	24	C	1.46
S17	24	C	1.56
S17	24	C	1.27
DSM 6208	0	As V	0.06
DSM 6208	0	As V	0.05
DSM 6208	0	As V	0.09
DSM 6208	0	As V	0.08
DSM 6208	3	As V	0.82
DSM 6208	3	As V	0.65
DSM 6208	3	As V	0.81
DSM 6208	3	As V	0.87
DSM 6208	6	As V	1.35
DSM 6208	6	As V	1.15
DSM 6208	6	As V	1.29
DSM 6208	6	As V	1.37

DSM 6963	6	As V	0.23
DSM 6963	6	As V	0.2
DSM 6963	6	As V	0.16
DSM 6963	6	As V	0.13
DSM 6963	6	As V	0.18
DSM 6963	6	As V	0.2
DSM 6963	6	As V	0.2
DSM 6963	9	As V	0.26
DSM 6963	9	As V	0.23
DSM 6963	9	As V	0.28
DSM 6963	9	As V	0.2
DSM 6963	9	As V	0.24
DSM 6963	9	As V	0.3
DSM 6963	9	As V	0.24
DSM 6963	12	As V	0.27
DSM 6963	12	As V	0.4
DSM 6963	12	As V	0.36
DSM 6963	24	As V	0.4
DSM 6963	24	As V	0.55
DSM 6963	24	As V	0.77
DSM 6963	0	As III	0.18
DSM 6963	0	As III	0.16
DSM 6963	0	As III	0.05
DSM 6963	0	As III	0.05
DSM 6963	0	As III	0.06
DSM 6963	0	As III	0.07
DSM 6963	3	As III	0.19
DSM 6963	3	As III	0.19
DSM 6963	3	As III	0.24
DSM 6963	3	As III	0.26

DSM 6208	9	As V	1.5
DSM 6208	9	As V	1.46
DSM 6208	9	As V	1.49
DSM 6208	9	As V	1.54
DSM 6208	12	As V	1.48
DSM 6208	12	As V	1.45
DSM 6208	12	As V	1.6
DSM 6208	18	As V	1.45
DSM 6208	18	As V	1.55
DSM 6208	18	As V	1.62
DSM 6208	24	As V	1.47
DSM 6208	24	As V	1.51
DSM 6208	24	As V	1.65
DSM 6208	0	As III	0.07
DSM 6208	0	As III	0.05
DSM 6208	0	As III	0.08
DSM 6208	0	As III	0.1
DSM 6208	3	As III	0.26
DSM 6208	3	As III	0.29
DSM 6208	3	As III	0.26
DSM 6208	3	As III	0.25
DSM 6208	6	As III	0.35
DSM 6208	6	As III	0.36
DSM 6208	6	As III	0.36
DSM 6208	6	As III	0.31
DSM 6208	9	As III	0.34
DSM 6208	9	As III	0.42
DSM 6208	9	As III	0.5
DSM 6208	9	As III	0.45
DSM 6208	12	As III	0.4

DSM 6963	3	As III	0.24
DSM 6963	3	As III	0.25
DSM 6963	6	As III	0.32
DSM 6963	6	As III	0.34
DSM 6963	6	As III	0.76
DSM 6963	6	As III	0.6
DSM 6963	6	As III	0.6
DSM 6963	6	As III	0.83
DSM 6963	9	As III	0.83
DSM 6963	9	As III	1.02
DSM 6963	9	As III	1.07
DSM 6963	9	As III	0.76
DSM 6963	9	As III	0.84
DSM 6963	9	As III	1.1
DSM 6963	12	As III	1.05
DSM 6963	12	As III	1.21
DSM 6963	12	As III	1.33
DSM 6963	12	As III	1
DSM 6963	12	As III	1.11
DSM 6963	12	As III	1.27
DSM 6963	18	As III	1.6
DSM 6963	18	As III	1.2
DSM 6963	18	As III	1.3
DSM 6963	18	As III	1.41
DSM 6963	24	As III	1.7
DSM 6963	24	As III	1.31
DSM 6963	24	As III	1.61
DSM 6963	24	As III	1.81

DSM 6208	12	As III	0.53
DSM 6208	18	As III	0.4
DSM 6208	18	As III	0.55
DSM 6208	18	As III	0.51
DSM 6208	18	As III	0.38
DSM 6208	24	As III	0.38
DSM 6208	24	As III	0.65
DSM 6208	24	As III	0.38
DSM 6208	24	As III	0.35
DSM 6208	0	C	0.1
DSM 6208	0	C	0.15
DSM 6208	0	C	0.11
DSM 6208	3	C	0.51
DSM 6208	3	C	0.41
DSM 6208	3	C	0.36
DSM 6208	6	C	1.18
DSM 6208	6	C	1.25
DSM 6208	6	C	1.07
DSM 6208	9	C	1.43
DSM 6208	9	C	1.56
DSM 6208	9	C	1.32
DSM 6208	12	C	1.55
DSM 6208	12	C	1.67
DSM 6208	12	C	1.56
DSM 6208	15	C	1.54
DSM 6208	15	C	1.69
DSM 6208	15	C	1.61
DSM 6208	18	C	1.58
DSM 6208	18	C	1.69
DSM 6208	18	C	1.63
DSM 6208	21	C	1.58

DSM 6208	21	C	1.72
DSM 6208	21	C	1.62
DSM 6208	24	C	1.54
DSM 6208	24	C	1.75
DSM 6208	24	C	1.68

2.2. <u>Biofilm production Data</u>

You should know the following information about the Dynamic data table of biofilm production measurements.

1- The two tables of data arranged to the left and right of the reading frame correspond to the strains *Exiguobacterium* sp. S17 (S17) and *Acinetobacter* sp. Ver3 (Ver3), respectively.
2- The second line consist of labels.
3- The first column consists in the kind of support material; Glass or Polypropylene.
4- The second columns consist in the arsenic oxidative status; “C” for cultures treated without arsenic, “As V” for arsenate and “As III” for arsenite.
5- The third column consist the arsenic doses applied to the cultures.
6- The fourth column consist in the biofilm production measures based on the read at DO 570 nm of the solubilized crystal violet dye.

S17			
**Support**	**As status**	**Doses**	**biofilm**
Glass	C	0	0.25
Glass	C	0	0.29
Glass	C	0	0.29
Glass	C	0	0.17
Glass	As V	50 mM	0.46
Glass	As V	50 mM	0.40
Glass	As V	50 mM	0.43
Glass	As V	50 mM	0.54
Glass	As V	150 mM	0.40
Glass	As V	150 mM	0.38
Glass	As V	150 mM	0.33
Glass	As V	150 mM	0.44
Glass	As V	250 mM	0.31
Glass	As V	250 mM	0.30
Glass	As V	250 mM	0.36

Ver3			
**Support**	**As status**	**Doses**	**biofilm**
Glass	C	0	0.32
Glass	C	0	0.21
Glass	C	0	0.3
Glass	As V	50 mM	0.13
Glass	As V	50 mM	0.15
Glass	As V	50 mM	0.085
Glass	As V	150 mM	0.112
Glass	As V	150 mM	0.19
Glass	As V	150 mM	0.101
Glass	As V	250 mM	0.23
Glass	As V	250 mM	0.16
Glass	As V	250 mM	0.187
Glass	As V	350 mM	0.2
Glass	As V	350 mM	0.084
Glass	As V	350 mM	0.083

Glass	As V	250 mM	0.47
Glass	As V	350 mM	0.17
Glass	As V	350 mM	0.18
Glass	As V	350 mM	0.17
Glass	As V	350 mM	0.11
Glass	As III	2,5 mM	0.41
Glass	As III	2,5 mM	0.35
Glass	As III	2,5 mM	0.39
Glass	As III	2,5 mM	0.46
Glass	As III	7,5 mM	0.22
Glass	As III	7,5 mM	0.19
Glass	As III	7,5 mM	0.28
Glass	As III	7,5 mM	0.20
Glass	As III	12,5 mM	0.05
Glass	As III	12,5 mM	0.03
Glass	As III	12,5 mM	0.08
Polypropylene	C	0	0.05
Polypropylene	C	0	0.01
Polypropylene	C	0	0.14
Polypropylene	C	0	0.11
Polypropylene	As V	50 mM	0.09
Polypropylene	As V	50 mM	0.02
Polypropylene	As V	50 mM	0.10
Polypropylene	As V	50 mM	0.11
Polypropylene	As V	150 mM	0.02
Polypropylene	As V	150 mM	0.12
Polypropylene	As V	150 mM	0.02
Polypropylene	As V	150 mM	0.09
Polypropylene	As V	250 mM	0.02
Polypropylene	As V	250 mM	0.10
Polypropylene	As V	250 mM	0.04
Polypropylene	As V	250 mM	0.09
Polypropylene	As V	350 mM	0.04
Polypropylene	As V	350 mM	0.01
Polypropylene	As V	350 mM	0.01
Polypropylene	As V	350 mM	0.07
Polypropylene	As III	2,5 mM	0.11
Polypropylene	As III	2,5 mM	0.16
Polypropylene	As III	2,5 mM	0.02
Polypropylene	As III	2,5 mM	0.08
Polypropylene	As III	7,5 mM	0.01
Glass	As III	2,5 mM	0.116
Glass	As III	2,5 mM	0.1425
Glass	As III	2,5 mM	0.112
Glass	As III	7,5 mM	0.101
Glass	As III	7,5 mM	0.101
Glass	As III	7,5 mM	0.098
Glass	As III	12,5 mM	0.02
Glass	As III	12,5 mM	0.1
Glass	As III	12,5 mM	0.02
Polypropylene	C	0	0.12
Polypropylene	C	0	0.106
Polypropylene	C	0	0.123
Polypropylene	As V	50 mM	0.135
Polypropylene	As V	50 mM	0.109
Polypropylene	As V	50 mM	0.107
Polypropylene	As V	150 mM	0.09
Polypropylene	As V	150 mM	0.088
Polypropylene	As V	150 mM	0.086
Polypropylene	As V	250 mM	0.087
Polypropylene	As V	250 mM	0.086
Polypropylene	As V	250 mM	0.083
Polypropylene	As V	350 mM	0.074
Polypropylene	As V	350 mM	0.074
Polypropylene	As V	350 mM	0.069
Polypropylene	As III	2,5 mM	0.14
Polypropylene	As III	2,5 mM	0.137
Polypropylene	As III	2,5 mM	0.129
Polypropylene	As III	7,5 mM	0.1
Polypropylene	As III	7,5 mM	0.093
Polypropylene	As III	7,5 mM	0.086
Polypropylene	As III	12,5 mM	0.02
Polypropylene	As III	12,5 mM	0.01
Polypropylene	As III	12,5 mM	0.01

Polypropylene	As III	7,5 mM	0.02
Polypropylene	As III	7,5 mM	0.10
Polypropylene	As III	7,5 mM	0.08
Polypropylene	As III	12,5 mM	0.00
Polypropylene	As III	12,5 mM	0.00
Polypropylene	As III	12,5 mM	0.00

2.3. Cell clumping analysis data

You should know the following information about the dynamic data table of cell clumping analysis.

1- The first line consists of labels.
2- The first column contains the labels (in numbers) of the images used to average the probability of cellular clumping (see the next section). The image tagged with a script was not included in the picture of the next section since that figure is arrange in set of four images.
3- The second column contains the treatments labels in which the image was sampled.
4- The third column contains the number of individual cells counted in the sampled.
5- The fourth column contains the total number of cells present in the sample.
6- The fifth column contains the measures of the probability of cellular clumping.

Picture	Treatment	Ind. Cells	Total Cells	P
1	Control	214	651	0.670198675
2	Control	175	738	0.761565836
3	Control	430	979	0.560073937
4	Control	127	310	0.59
5	50 mM	359	1332	0.73048048
6	50 mM	252	1440	0.825
7	50 mM	257	1207	0.787075394
8	50 mM	285	1059	0.730878187
9	150 mM	567	1647	0.655737705
10	150 mM	531	1327	0.599849284
11	150 mM	194	953	0.796432319
12	150 mM	174	1050	0.834285714
13	350 mM	98	372	0.73655914
14	350 mM	86	210	0.59047619
15	350 mM	79	429	0.815850816
16	350 mM	35	275	0.872727273

17	2.5 mM	270	728	0.629120879
18	2.5 mM	206	397	0.481108312
19	2.5 mM	155	306	0.493464052
20	2.5 mM	190	523	0.633911368
–	2.5 mM	329	532	0.381578947
21	7.5 mM	80	115	0.304347826
22	7.5 mM	153	195	0.215384615
23	7.5 mM	140	183	0.234972678
24	7.5 mM	305	498	0.387550201

2.4. Imaging data

The images provided in this section were used to calculate the probability of cellular clumping. Each image is labelled with a number that corresponds to a P value calculated for that image in the above data set (see Cell clumping analysis data set).

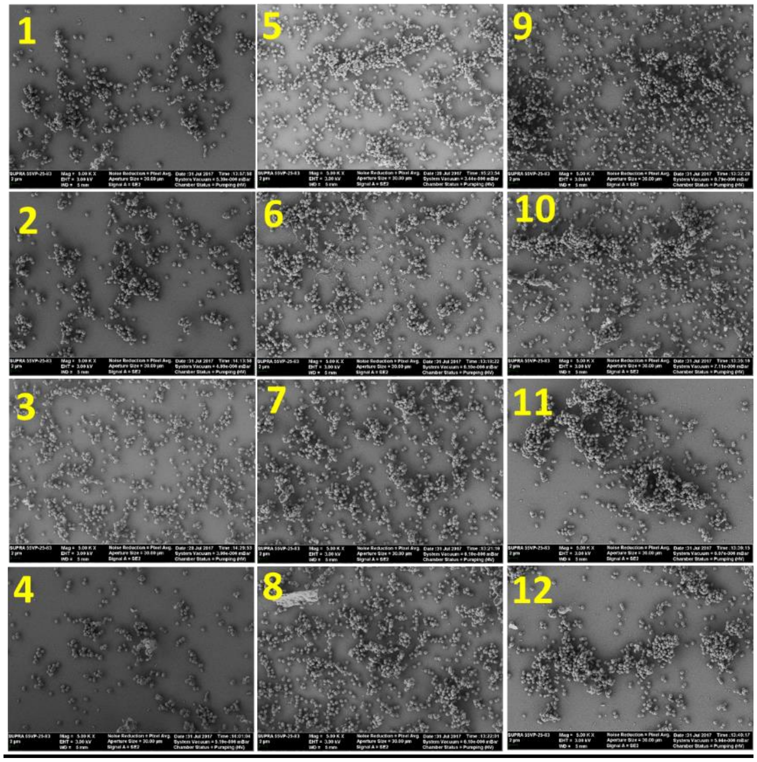

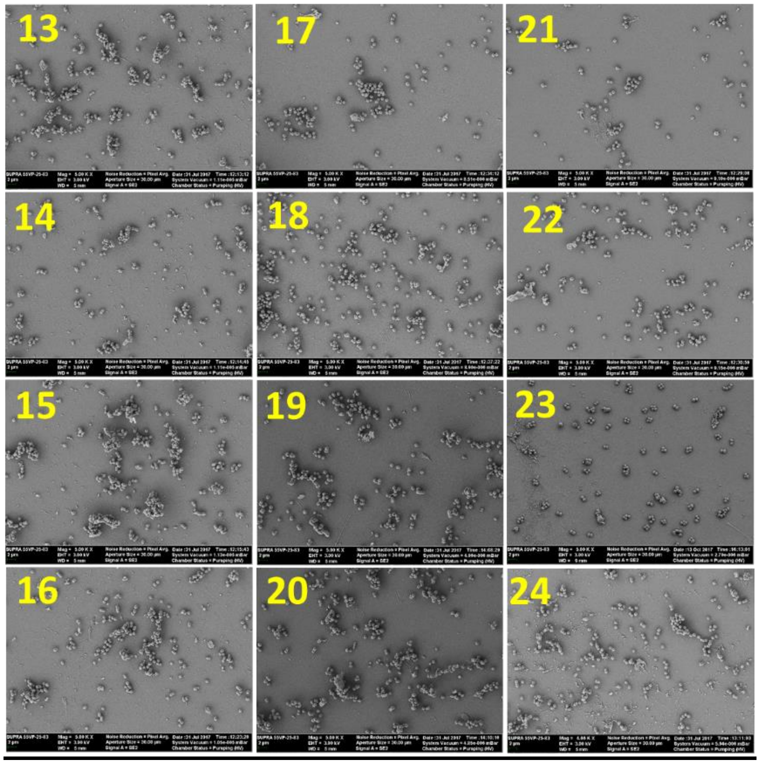

3. **Method detail**

Here we provide a detailed protocol, including materials and a step by step procedure to acquired high quality SEM biofilm images. Also, the first six steps are shared with the biofilm quantification protocol using colorimetric methods. In this sense, users can acquire both SEM measurements as well biofilm quantification from the same sample, improving the information.

For the methodology provided here, biofilm was produced in LB medium from *Exiguobacterium* sp. S17, an aerobic Gram-positive bacillus. However, the protocol is applicable for many specimens of interest, especially for those which tend to form mature biofilms at the air-medium interface.

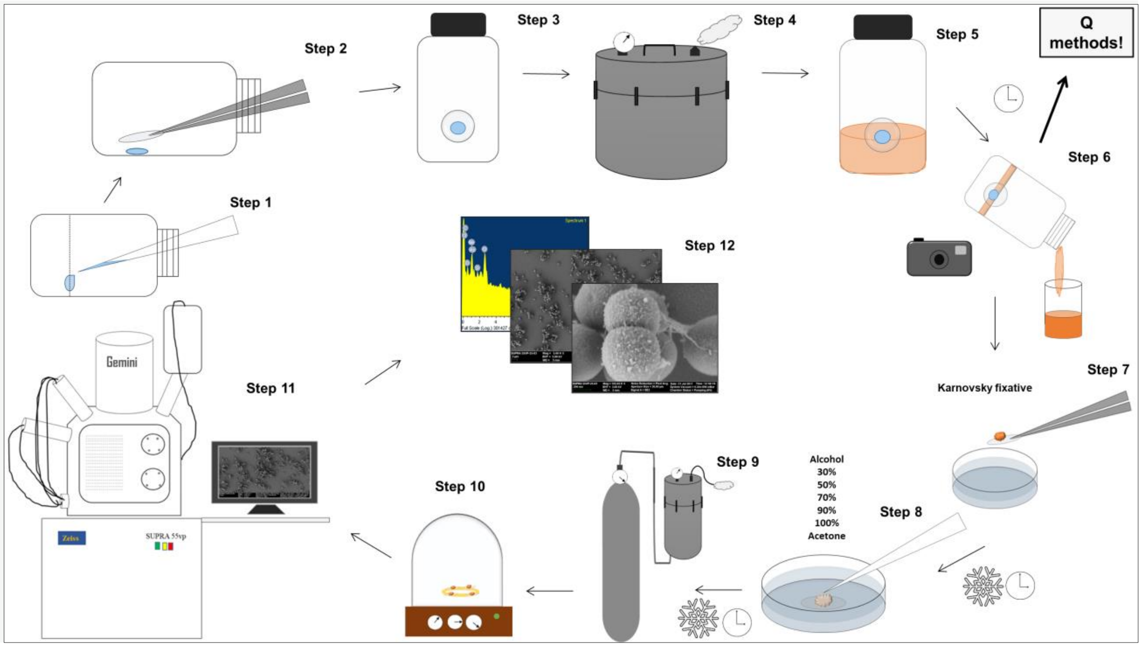

Graphic depiction of the protocol used in the scanning electron microscopy assay.

3.1. Materials

1. Graduated cylinder
2. Falcon tube
3. Flask
4. Pen
5. Nail polish
6. SEM Microscopy glass dishes (10 mm diameter) (slides)
7. Vortex
8. Paper napkin
9. Ethanol 98%
10. Forceps
11. Karnovsky fixative solution
12. Pasteur pipettes
13. Glass or polypropylene wells
14. Autoclave
15. Alcohol 30%, 50%, 70%, 90%, 100%
16. Acetone
17. Photo camera
18. Critical point drier apparatus
19. Gold coated apparatus

3.2. Procedure

Step 1. Add a desired volume1 of distilled water into a clean flask with a graduated container such as a graduated cylinder or a falcon tube, and mark the air-liquid interface with a pen (punctuated line). Use the marked flask as reference to mark all the flask of your experiment. Then apply a drop of nail polish2 on the mark on the inner surface of the dry flasks and let dry for 5 minutes. In our experiment, we add 20 ml into a 100 ml flask.

Note 1: The same culture volume that you will use in your experiment.

Note 2: The nail polish was the best glue we proved, as it pasted the SEM microscopy slide, withstands the high temperatures of the autoclave without peeling off and easily peels off without breaking the SEM microscopy slide.

Step 2. Clean the slides by placing them into a falcon tube, add 20 ml of 98% ethanol and vortex for 1 minute. Remove the slides from the falcon tube and let them dry on a paper napkin. Keep the slides covered with a lid.

With clean forceps attach the slide on the nail polish drop. Keep flasks horizontally for 20 minutes until the slides are glued.

Step 3 and 4. Close the flasks with their respective caps and autoclave for 15 minutes. After autoclaving, re-label the flasks of each treatment neatly.

Step 5. Under sterile conditions, add the pre-established volume of your culture at a desired optical density into the flask. Ensure that the air-medium interface is at the half or lower half of the slide. If your strain needs agitation, adjust the rpm of your agitator so that the culture does not exceed the height of the slide.

In our experiment, we cultured Exiguobacterum sp.S17 at OD 600 nm 0.8 and at 120 rpm for 48 hours

Step 6. At the end of the incubation period, the mature biofilm will have formed as a ring at the air-medium interface and on the slide. Take a picture of the flask of each treatment 3 before discarding the culture into a breaker. Rinse the flask with sterilized physiological solution three times. With a forceps push gently the slide and release it. The remaining biofilm in the flasks can be quantified through multiple colorimetric methods (Peeters et al., 2008).

Nota 3: Establish a place in your laboratory with good lighting and cleaning for taking pictures. We suggest the interior of the laminar flow cabin. Place the flask at an established distance from the camera and take the pictures.

Step 7. Put the slide into a well containing Karnovsky fixative solution4 making sure that the sample of biofilm is in the upper face of the slide. Ensure that the solution covers the slide. Tightly close the well with parafilm and store in the refrigerator door for 24-168 hours.

Note 4: To improve the procedure, use wells of materials not soluble in acetone such as glass or polypropylene.

Step 8. Take the sample from the refrigerator, uncover the well and dehydrate the sample with alcohols. Use a Pasteur pipette to remove Karnovsky fixative solution and sequentially add 3 ml of alcohol 30%, 50%, 70%, 90%, 100%, for 10 minutes each. Replace alcohol 100% with acetone and store in the refrigerator for 24 hours.

Step 9. Finish drying the samples with the Critical Point Dried technique using the traditional liquid carbon dioxide. This procedure quickly replaces the acetone content with carbon dioxide, preventing the collapse of the cells in the biofilm sample. In our experiment, we use a Denton Vacumm DCP-1 Critical Point Dryer Apparatus.

Step 10. Coat the samples with a gold pellicle of 10 nm. We perform this procedure with a JeoL Fine Coat Ion Sputter (JFC-1100) adjusted at 1.2 KV and 2.5 mA. After the gold coated procedure, the samples are ready to be seen with SEM.

Step 11. Mount the samples on the SEM stub and star your SEM analysis. We use a Zeiss Supra 55 vp scanning electron microscope setting EHT voltage at 3.00 Kv and a work distance (WD) of 5 mm.

Step 12. Identify cell distribution patterns, channels between cells and cellular secondary structures that promote cell aggregation. If you treated cultures with toxic compounds such as heavy metals, perform an EDS analysis to corroborate the presence of these ions in the sample.
